# Investigation of Layered Structure Formation in MgB_2_ Wires Produced by the Internal Mg Coating Process under Low and High Isostatic Pressures

**DOI:** 10.3390/ma17061362

**Published:** 2024-03-16

**Authors:** Daniel Gajda, Michał Babij, Andrzej Zaleski, Doğan Avci, Fırat Karaboga, Hakan Yetis, Ibrahim Belenli, Tomasz Czujko

**Affiliations:** 1 Institute of Low Temperature and Structure Research, Polish Academy of Sciences (PAS), Okólna 2, 50-422 Wroclaw, Poland; m.babij@intibs.pl (M.B.); a.zaleski@intibs.pl (A.Z.); 2 Quatum Metrology Laboratory, National Metrology İnstitute TÜBİTAK, 41470 Kocaeli, Turkey; davci.0209@gmail.com; 3 Mehmet Tanrikulu Vocational School of Health Services, Bolu Abant Izzet Baysal University, 14030 Bolu, Turkey; karabogafirat@ibu.edu.tr; 4 Department of Physics, Bolu Abant Izzet Baysal University, 14280 Bolu, Turkey; hknyetis@gmail.com (H.Y.); belenli_i@ibu.edu.tr (I.B.); 5 Institute of Materials Science and Engineering, Military University of Technology, Kaliskiego 2, 00-908 Warsaw, Poland

**Keywords:** layered structure, MgB_2_ wires, HIP process

## Abstract

Currently, MgB_2_ wires made by the powder-in-tube (PIT) method are most often used in the construction and design of superconducting devices. In this work, we investigated the impact of heat treatment under both low and high isostatic pressures on the formation of a layered structure in PIT MgB_2_ wires manufactured using the Mg coating method. The microstructure, chemical composition, and density of the obtained superconductive wires were investigated using scanning electron microscopy (SEM) with an energy-dispersive X-ray spectroscopy (EDS) analyzer and optical microscopy with Kameram CMOS software (version 2.11.5.6). Transport measurements of critical parameters were made by using the Physical Property Measurement System (PPMS) for 100 mA and 19 Hz in a perpendicular magnetic field. We observed that the Mg coating method can significantly reduce the reactions of B with the Fe sheath. Moreover, the shape, uniformity, and continuity of the layered structure (cracks, gaps) depend on the homogeneity of the B layer before the synthesis reaction. Additionally, the formation of a layered structure depends on the annealing temperature (for Mg in the liquid or solid-state), isostatic pressure, type of boron, and density of layer B before the synthesis reaction.

## 1. Introduction

Currently, MgB_2_ wires made by the powder-in-tube (PIT) method are most often used in the construction and design of superconducting magnets for magnetic energy storage (SMES), wind turbines, magnetic resonance imaging (MRI), cables and electric motors [1,2,3]. This is due to the low anisotropy, inexpensive components, high critical temperature, high upper magnetic field, and low mass density of these materials [4,5,6,7]. These advantages are more important when the production of monocore or multi-filament PIT MgB_2_ wires with lengths longer than 1 km is desired.

However, PIT MgB_2_ wires have several disadvantages, such as a large number of voids, poor connections between grains, low density of pinning centers, low irreversible magnetic field, and large grains [8,9,10,11,12,13,14]. Current research has shown that doping allows for increasing the density of pinning centers [15,16,17,18,19,20,21]. Heating under pressure increases the number of connections between grains, reduces the amount of voids, and creates pinning centers [22,23,24]. Kim et al. [25] have shown that the synthesis reaction depends on the type of boron (e.g., crystalline or amorphous), purity, and size of the boron grains. Further research has shown that a higher density of unreacted Mg + 2B material also enhanced the number of grain connections, reduced the size and number of voids, and accelerated the synthesis reactions [26,27].

Previous studies have shown that the layered (fiber) structure has a significant impact on the critical transport parameters, especially the critical current density [28,29,30,31]. Uchiyama et al. [28] have indicated that a fiber (layer) structure was formed as a result of annealing MgB_2_ in the solid Mg-state. They noted that finer fibers allowed for a higher transport-critical current density in MgB_2_ wires. Additionally, heat treatment in the liquid state of Mg was shown to cause damage to MgB_2_ fibers [28]. Susner et al. [29] have shown that cold drawing (CD) leads to elongation and a decrease in the thickness of Mg grains and textures the morphology of MgB_2_ material after heat treatment. They suggested that elongated Mg grains affect the orientation and shape of the boron layer. Moreover, Susner et al. [29] have indicated that MgB_2_ fibers were separated by voids created by the diffusion of Mg into the B layer. Mroczek et al. [30] have shown that annealing at 630 °C and 650 °C caused the formation of an island structure with a small number of connections in PIT MgB_2_ wires [30]. Furthermore, they showed that annealing at 700 °C resulted in the formation of layered structures in PIT MgB_2_ wires [30]. Other results have indicated that thermal treatment at 700 °C under isostatic pressures of 0.3 GPa and 1.1 GPa also caused the formation of a layered structure. On the other hand, Mroczek et al. [30] have also shown that an isostatic pressure of 0.8 GPa and an annealing temperature of 700 °C did not result in the formation of a layered structure [30]. This difference may be considered a result of the transition of Mg from the solid to the liquid state. The layered structure after the HIP process allowed for a very high transport-critical current density at 4.2 K and 20 K [30]. The latest results obtained for PIT MgB_2_ wires with isotope B (^11^B) show that annealing at 700 °C and 740 °C for 40 min formed small grains and weak connections between grains and did not form a layered structure, but that annealing at 800 °C gave rise to the formation of a layered structure in the PIT Mg^11^B_2_ wires [31]. These results show that a dense layer structure allowed for a high transport current density to be achieved in PIT Mg^11^B_2_ wires [31], which is important for future fusion reactors. It was also shown that the multilayer thin films allow for the obtaining of a very high critical current density of 1000 A/mm^2^ at 12 T at 4.2 K [32].

In this work, we investigated the impact of heat treatment under both low and high isostatic pressures on the formation of a layered structure in PIT MgB_2_ wires manufactured using the Mg coating method. Moreover, our results unveiled the factors that influence the shape, length, uniformity, and density of the layered structure. Additionally, we demonstrated the influence of heating under high isostatic pressure on crucial parameters, such as the critical temperature, irreversible magnetic field, and upper critical field.

## 2. Materials and Methods

**Figure 1:**
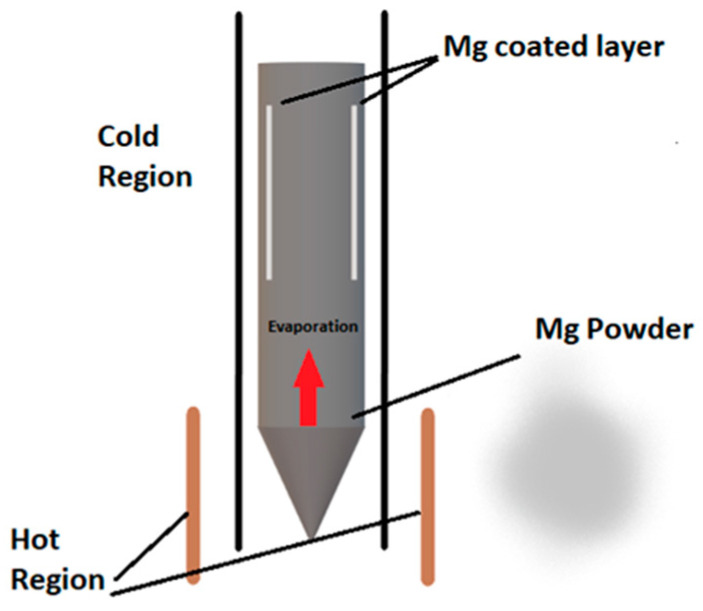
A schematic representation of the Mg coating process.

The MgB_2_ wires were made by using the powder-in-tube (PIT) and Mg coating methods [33]. In this study, the inner surface of an iron tube (outer/inner diameter: 12/9 mm) was coated with magnesium. A schematic representation of the Mg coating process is shown in Figure 1.

A small amount of Mg powder (PVZ, 99%, 325 mesh) was added to an iron tube (*l*_it_ = 250 mm). The tube was placed inside a long steel tube (*l*_st_ = 1800 mm) with a valve to heat the tube in a vertical furnace. The temperature of the furnace was increased to 600 °C under vacuum at a rate of 5 °C/min, and then the steel tube was kept at 600 °C for 15 min to complete the Mg coating process [33]. The stoichiometric Mg + 2B powder was ball milled and mixed for 3 h using a Retsch PM 100 (Retsch GmbH, Haan, Germany) planetary ball mill (rotation speed 200 rpm, ball to powder ratio 4), and then the mixture was filled into an internally Mg-coated iron tube using the PIT method. The boron powder mixture consisted of two distinct types of powders differing in terms of purity and particle size: semicrystalline boron (PVZ-B, 95–97% purity, and particle sizes <1 µm) and amorphous nanoboron (PVZ-B, 98.5% purity, and particle sizes <250 nm). These powders were used in equal masses [26]. The Fe/MgB_2_ wire was produced with a 0.81 mm diameter by using a cold drawing process (filling factor of superconducting material—40%).

The tubes prepared in this way were annealed at a temperature of 700 °C for 40 min under varying argon pressures of 0.1 MPa and 1.1 GPa (Table 1).

Following heat treatment, the wires were cut into short pieces, which were subsequently polished to examine the structural morphology in both longitudinal and cross sections. Analyses of the microstructure and composition were performed by using scanning electron microscopy SEM; FEI Nova Nano SEM 230 (Hillsboro, OR, USA) and a Quanta 3D FEG (Quanta, Hillsboro, OR, USA).

Additionally, cross-sections were taken by using an optical microscope (Olympus GX41, Olympus Corporation, Tokyo, Japan). The porosity (density-color map) analysis of the samples was carried out by using software (Kameram CMOS, version 2.11.5.6). The colors were assigned based on the intensity of the reflected light, with denser structures reflecting significantly more light than weaker structures.

Transport measurements of critical parameters were made by using the Physical Property Measurement System (PPMS) for 100 mA and 19 Hz in a perpendicular magnetic field. The critical parameters were determined based on the criteria of 10% for *B*_irr_, 50% for *T*_c_, and 90% for *B*_c2_.

## 3. Results and Discussion

In this paper, for the first time, the impact of thermal treatment under high isostatic pressure on the morphology and critical parameters of PIT MgB_2_ wires made by using the Mg coating process is presented. We demonstrate that the Mg coating process significantly reduced the formation of intermetallic phases between the superconducting MgB_2_ material and the iron shield, e.g., iron borides. These phases were also observed in Fe-sheathed MgB_2_ wires [27]. These phases are unfavorable because they reduce the amount of the superconducting phase, lengthen the path of current penetration into the superconducting material, and change the mechanical properties of the PIT MgB_2_ wires in a negative manner. Additionally, our results indicate that the Mg coating process may replace diffusion barriers (e.g., Nb and ex situ MgB_2_) in the future [34,35]. This approach will reduce the price of PIT MgB_2_ wires and simplify their production. Moreover, Mg coating will allow for homogeneous structures of MgB_2_ material to be obtained in wires longer than 1 km, which is necessary for superconducting coils [1,3].

### 3.1. Structural Analysis of MgB_2_ Wires

**Figure 2:**
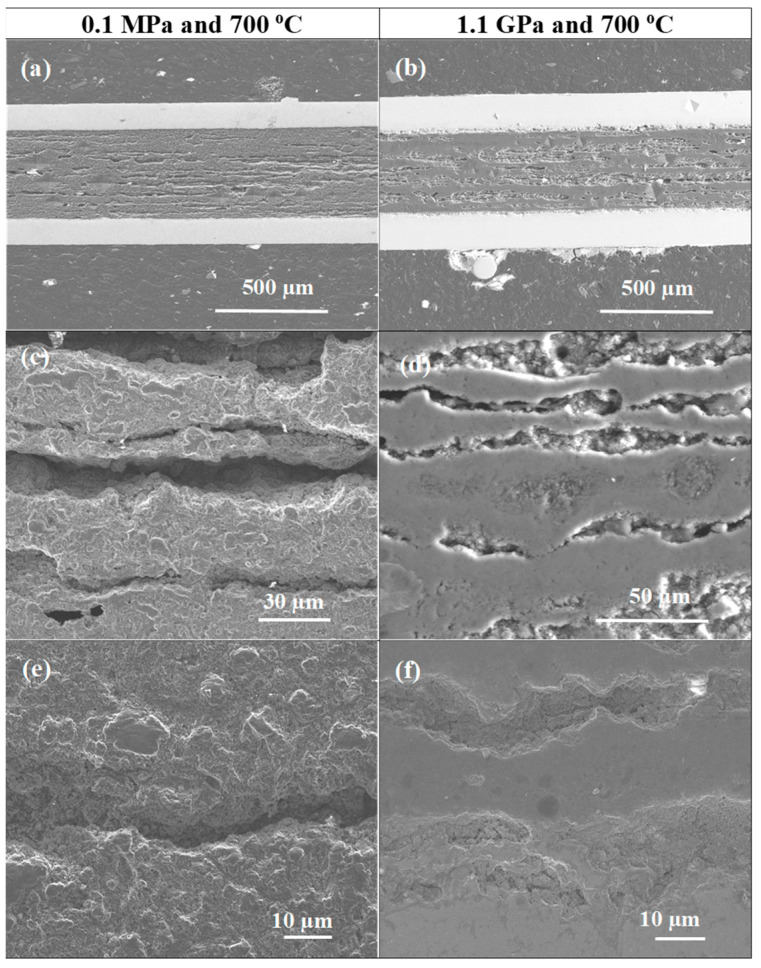
SEM images and longitudinal sections of single-core undoped MgB_2_ wires (**a**,**c**,**e**) sample heat treated under low isostatic pressure (0.1 MPa) and (**b**,**d**,**f**) sample annealed under high isostatic pressure (1.1 GPa).

**Figure 3:**
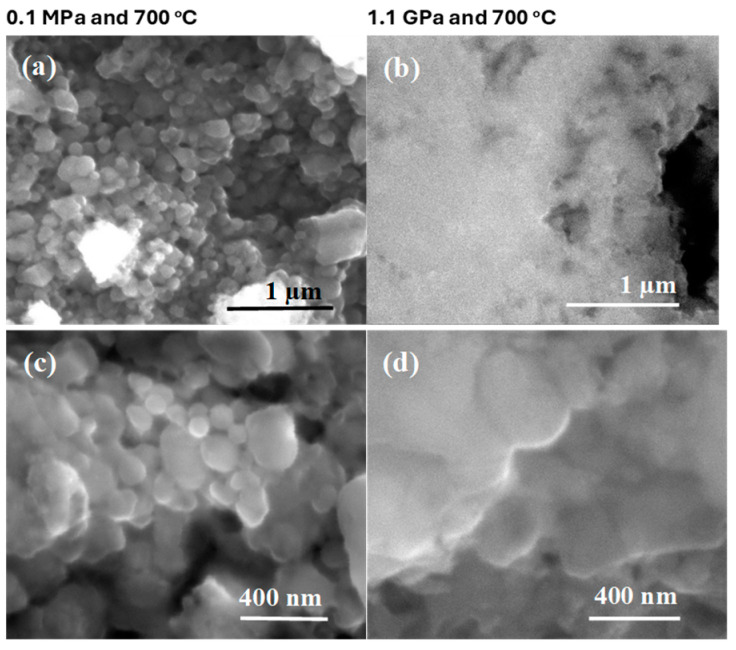
High-magnification longitudinal sections of single-core undoped MgB_2_ wires (**a**,**c**) annealed under low isostatic pressure (0.1 MPa) and (**b**,**d**) annealed under high isostatic pressure (1.1 GPa).

Figure 2 and Figure 3 show the morphologies of samples A and B, which were heated and treated under low and high isostatic pressures. The longitudinal section results show that samples heat treated under low and high isostatic pressures have different layered structures.

Sample A (0.1 MPa) has a layered structure composed of small grains, high porosity, short connections between grains, and low density. However, sample B was (Figure 2) heat treated under high isostatic pressure and has a layered structure consisting of two regions. One has a high density, and the other has a low density (Figure 2d). Moreover, in Figure 2a–d, we see large voids that were created as a result of the diffusion of Mg into layer B. This indicated that the superconducting phase MgB_2_ was formed. Jung et al. [36] have indicated that Mg + 2B material could shrink by 25% during the formation of the superconducting MgB_2_ phase.

The pictures taken at high magnification in Figure 3 show that sample A has a grain size of up to 200 nm and a void size of up to 400 nm. However, sample B has a uniform and homogeneous structure without any voids (Figure 3b,d).

**Figure 4:**
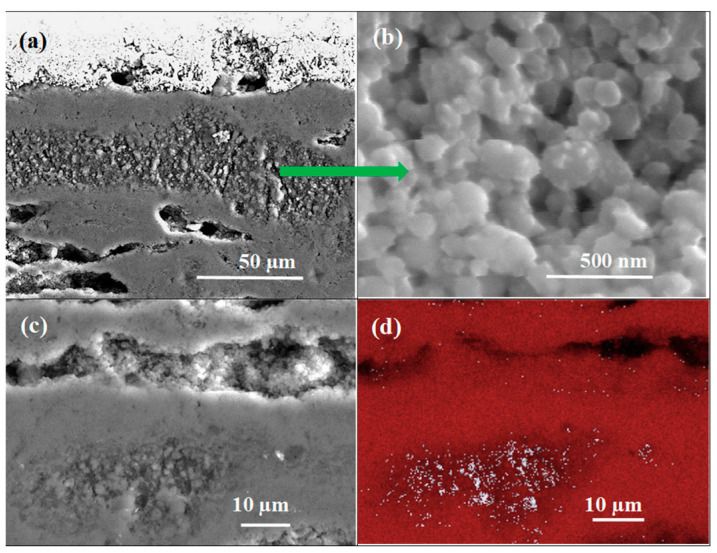
Longitudinal section of single-core undoped MgB_2_ heated under high isostatic pressure (1.1 GPa): (**a**–**c**) secondary electron (SE) and (**d**) energy-dispersive X-ray spectroscopy (EDS) analysis; red indicates Mg, and white indicates B.

The analyses given in Figure 4 indicate that the low-density region in sample B is formed by unreacted B, with a grain size of up to 250 nm.

**Figure 5:**
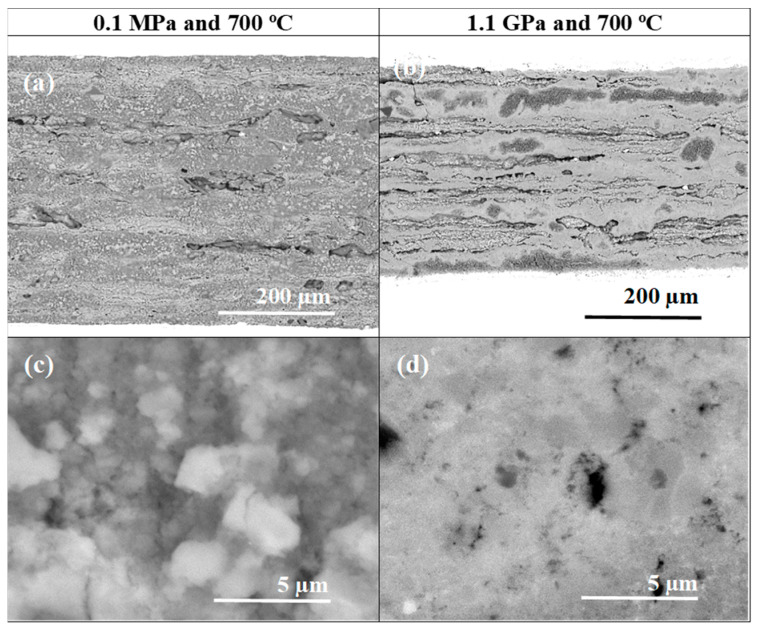
Longitudinal section-backscattered electron (BSE) analysis of (**a**,**c**) sample A (0.1 MPa) and (**b**,**d**) sample B (1.1 GPa). Dark regions indicate B. Light regions indicate Mg.

The backscattered electron (BSE) analysis showed that sample A had a uniform MgB_2_ phase distribution and did not contain unreacted Mg or B (Figure 5a,c).

Furthermore, the BSE results (Figure 4b,d) show that sample B has a large amount of unreacted B and Mg. Figure 4 shows that unreacted B can appear in both layered and island structures. This result is very important because it reveals the morphology and distribution of the layers of unreacted B. Susner et al. [29] have shown that Mg diffused into the B layer and created a superconducting MgB_2_ phase and voids. Based on these results, it can be concluded that the unreacted B layers have the greatest influence on the shape, thickness, and distribution of the layered structure. This also indicates that interruptions in the layered structure and weak connections between layered structures result from the nonuniform distribution of B before the synthesis reaction. Our results indicate that further research should be devoted to obtaining a more uniform distribution of B layers and increasing the density of the unreacted B layer in PIT MgB_2_ wires. This approach ensures a more uniform layer structure, a greater number of connections between layers, and a higher critical current density.

**Figure 6:**
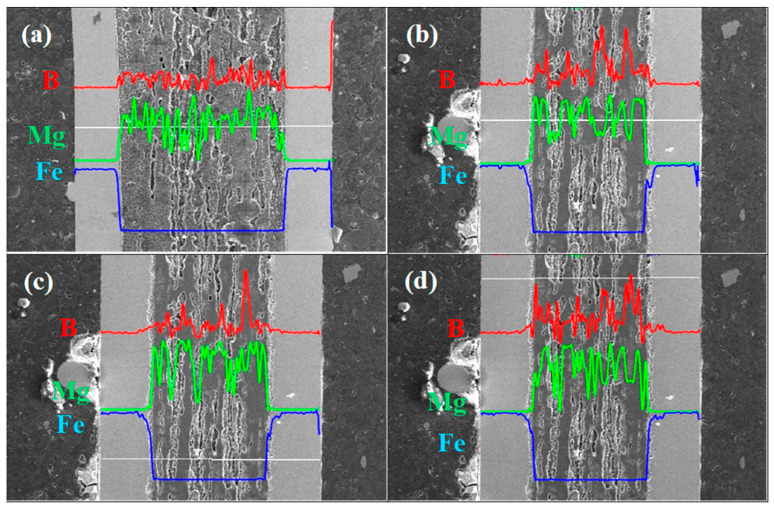
Linear analysis of the sample composition for longitudinal sections of (**a**) sample A (0.1 MPa) and (**b**–**d**) sample B (1.1 GPa). The red line denotes B, the green line shows Mg, and the blue line indicates the iron sheath (Fe).

The results in Figure 6a show that sample A has uniformly distributed B and Mg. This indicates that the superconducting MgB_2_ phase has a uniform structure in sample A. However, the results in Figure 6b–d show that sample B has regions with high B contents that are distributed nonuniformly in the wire. This also reveals a nonuniform distribution of the superconducting MgB_2_ phase and pure (unreacted) Mg.

**Figure 7:**
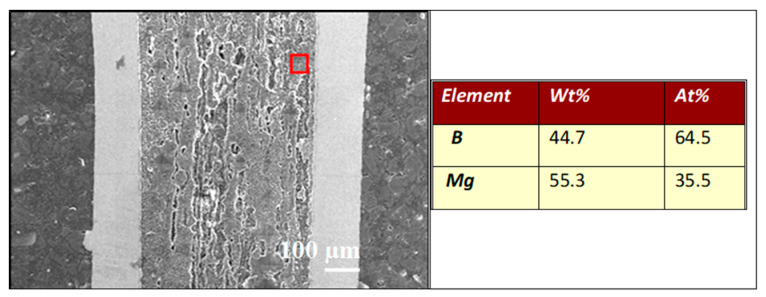
EDS analysis of the longitudinal section of sample A (0.1 MPa).

**Figure 8:**
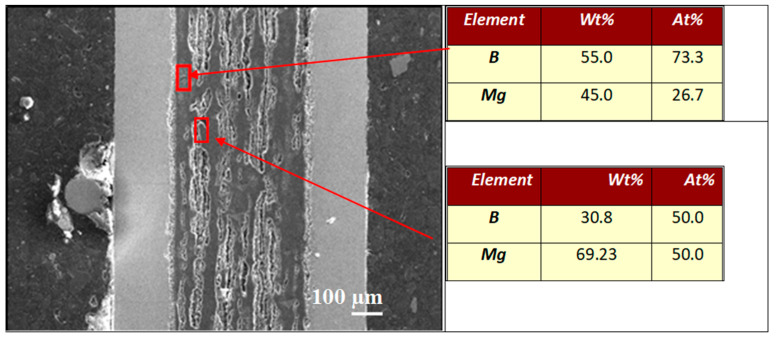
EDS analysis of the longitudinal section of sample B (1.1 GPa). The red square indicates the place for the EDS analysis was performed.

The EDS analysis in Figure 7 shows that sample A has a homogeneous distribution of Mg (35.5 at %) and B (64.5 at %), facilitating the homogenous formation of the MgB_2_ phase. The results in Figure 8 show that sample B has regions with excess B (73 at %—B, 27 at %—Mg) and others with equal amounts of Mg and B (50 at %—Mg and B). Sample B has three different regions: pure (unreacted) Mg, pure (unreacted) B, and the superconducting MgB_2_ phase.

The results obtained from the longitudinal section indicate that the layered structure of sample A, created under low isostatic pressure and in the liquid state of Mg, consists mainly of high-porosity MgB_2_. The results obtained for sample B show that the layered structure consists of three regions: pure Mg, pure B, and the superconducting MgB_2_ phase. The pure Mg and the superconducting MgB_2_ phase form high-density regions. However, low-density regions are formed due to the presence of pure B. Since an isostatic pressure of 1.1 GPa increases the melting point of Mg to 730 °C, Mg is in the solid state; therefore, unreacted Mg and B remain in the structure [6,37]. The process of Mg diffusion into the B layers slows down when Mg is in the solid-state phase. The second reason may be the use of crystalline boron with a grain size of 1 μm in sample A. Kim et al. [25] have indicated that crystalline boron with large grains reacted more slowly than nano amorphous boron (especially during reactions in the solid Mg state). Another factor influencing Mg diffusion is the density of the B layer. Li et al. have shown that the high density of the B layer significantly slowed the Mg diffusion process [38]. The results presented in [27] show that the solid-state Mg synthesis reaction at low isostatic pressure (630 °C, 0.1 MPa, 40 min) does not create any voids; large Mg particles and pure B remain. This indicates that the diffusion of Mg in the solid-state in the PIT MgB_2_ wires is very slow. On the other hand, based on our results, heat treatment under high isostatic pressure in the solid-state significantly accelerates the process of Mg diffusion into the B layers in PIT MgB_2_ wires. This is due to the large number of voids formed in sample B as a result of the diffusion of Mg to the B layer and the formation of the superconducting MgB_2_ phase. This also indicates that the synthesis reaction and Mg diffusion during the HIP process are more dependent on the pressure than the annealing temperature during the reaction in the solid-state phase of Mg.

**Figure 9:**
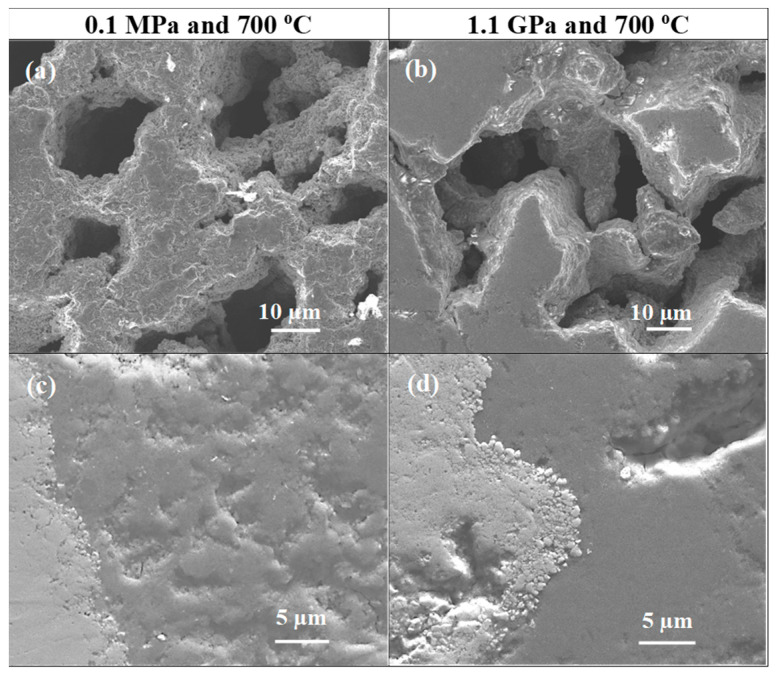
The SEM photo cross-sections of single-core undoped MgB2 wires (**a**,**c**) annealed under low isostatic pressure (0.1 MPa), (**b**,**d**) annealed under high isostatic pressure (1.1 GPa).

The cross-sectional images in Figure 9 show that sample A has a different morphology than sample B. Sample A has a granular structure with large and small voids. However, sample B has large voids and dense surfaces without voids.

**Figure 10:**
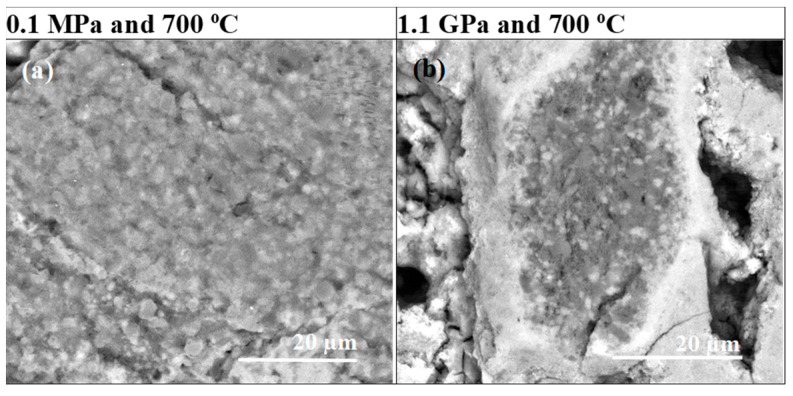
The cross-sections of MgB_2_ wires-backscattered electron (BSE) analysis (**a**,**b**) sample A (0.1 MPa) and sample B (1.1 GPa). Dark regions indicate B. Light regions indicate Mg.

**Figure 11:**
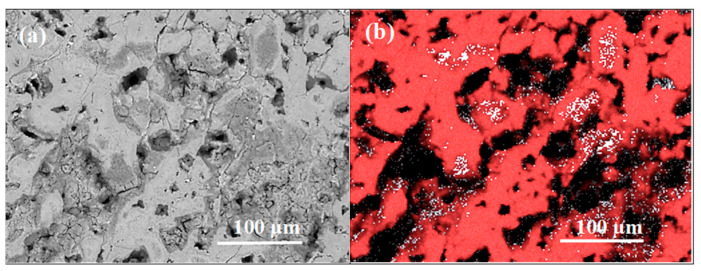
The cross-sections of sample B (1.1 GPa): (**a**) secondary electron (SE) and (**b**) energy dispersive X-ray spectroscopy (EDS) analysis. The red color indicates Mg, and the white color indicates B.

**Figure 12:**
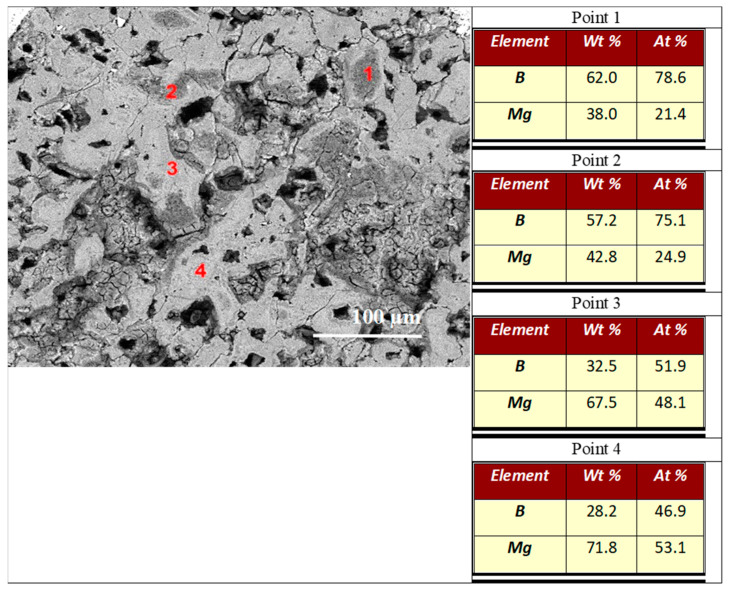
EDS analysis of cross-sections of sample B (1.1 GPa).

The backscattered electron (BSE) analysis of the cross section shows that sample A had a homogeneous material distribution and no pure B (Figure 10a). However, the BSE (Figure 10) and EDS (Figure 11) results show that sample B has a large amount of unreacted B. The EDS analysis of sample A shows that it has a homogeneous composition. However, sample B has two regions, one with an excess of B and one with an equal amount of Mg and B. The results for sample B in Figure 10b, Figure 11 and Figure 12 show that the regions with excess B have different shapes and surfaces. These results are very important because a layered structure consists of layers with different shapes (e.g., thickness and width). The cross-sectional examination revealed that sample A was a homogeneous superconducting material with a granular structure and a large number of pores (voids). However, the cross-section of sample B consists of three phases: the MgB_2_ superconducting phase, pure (unreacted) B, and pure (unreacted) Mg. The high-density regions have a large amount of Mg. However, the low-density regions are mainly composed of B. The apparent feature results shown in Figure 2 and Figure 12 reveal that samples A and B have similar longitudinal and cross-sectional compositions and morphologies. This indicates high uniformity in terms of the morphology of samples A and B.

**Figure 13:**
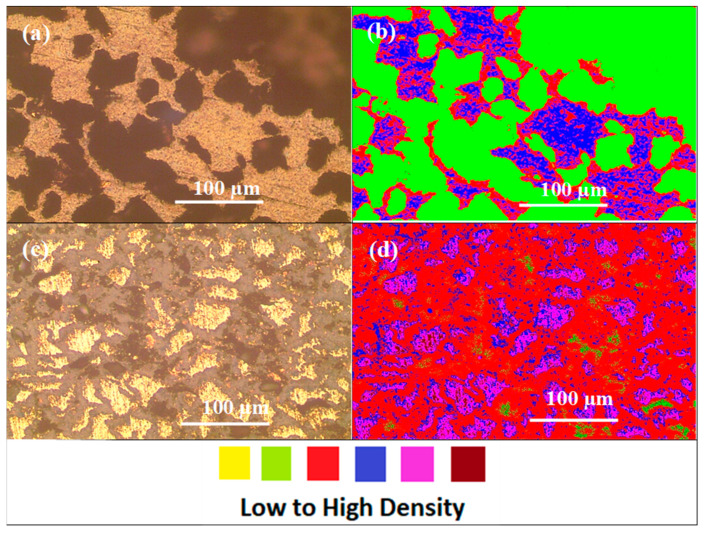
Morphological analysis via optical microscopy (**a**,**b**) cross-sections of sample A (0.1 MPa) and (**c**,**d**) cross-sections of sample B (1.1 GPa). The colors indicate the density of the MgB_2_ material. The low-density areas are yellow and green. Blue and pink indicate high density.

The images in Figure 13a,c were taken using an optical microscope. Sample B has a large number of small regions of high density; in contrast, sample A has large voids and large regions of low density. The EDS analysis shows that these regions were composed largely of pure Mg (Figure 5a, Figure 6, Figure 7, Figure 10a and Figure 12). Moreover, the results in Figure 13a show that sample A has a very uniform structure (similar porosity) of superconducting MgB_2_ material in the layers. Figure 13b,d shows the color maps for the porosity of the samples. The colors were defined according to the intensity of the reflected light. The dense structure exhibited a significantly greater light reflection (navy blue, pink and brown) than the weak structure (green, red) did. The EDS analyses of the cross-section and longitudinal section revealed that sample A mainly has the superconducting MgB_2_ phase without pure Mg or B. The navy blue and red colors in Figure 13b represent the MgB_2_ superconducting phase. The color mapping allows for the porosity analysis (density) of the layered structure and the connections between the layered structures. Figure 13b shows that the layered structure in sample A consists of dense regions (lower porosity) inside and low-density regions outside (higher porosity). Furthermore, the connections between the layers result in a significantly high porosity (low density). This indicates that there are fewer connections between individual superconducting MgB_2_ layers. The lack of proper connections at the junctions between superconducting layers reduces the transport critical current density in PIT MgB_2_ wires. These results indicate that the porosity of the interlayer connections should be reduced to achieve a higher transport critical current density in PIT MgB_2_ wires. These results show, for the first time, the importance of the morphology of the regions connecting the layered structures with each other for higher critical currents.

The results in Figure 13d show that sample B has a very heterogeneous morphology (low- and high-porosity regions). Figure 13d shows that sample B has a small number of navy blue regions and a large number of red regions. Dark blue regions are formed at the outer parts of dense regions (excess Mg–Figure 12). Based on the results given in Figure 4, Figure 5b, Figure 6b,c, Figure 8, Figure 10b, Figure 11 and Figure 12, it can be concluded that the HIP process allows us to obtain superconducting MgB_2_ material (in the dark blue regions) with a high density on the outer surface with excess Mg. This may suggest that the high isostatic pressure applied during the solid-state Mg synthesis reaction influences the formation and porosity of the MgB_2_ material, but the heating temperature does not.

### 3.2. Irreversible Magnetic Field Analysis for MgB_2_ Wires

**Figure 14:**
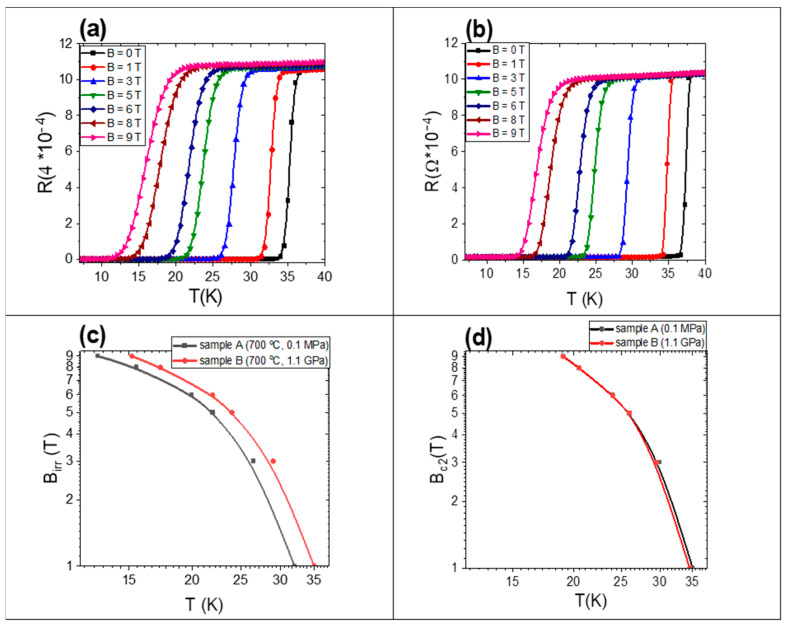
(**a**) Dependence of resistance on temperature for a sample heated under low isostatic pressure. (**b**) Dependence of resistance on temperature for a sample heated under high isostatic pressure. (**c**) Dependence of the irreversible magnetic field (*B*irr) on temperature. (**d**) Dependence of the upper magnetic field (*B*_c2_) on temperature.

The results presented in Figure 14a,b show that after the HIP process the sample has a lower resistance in the normal state by 6% than the sample heated under low isostatic pressure. This suggests that the HIP process creates more connections. Moreover, the R–T curves after the HIP process have a sharper transition to the resistive state than the R–T curves after heating under low isostatic pressure. This indicates that the MgB_2_ superconducting phase is more homogeneous after the HIP process.

Transport measurements indicated that both samples have similar critical temperatures of approximately 36 K. Sample B has a higher *B*_irr_ than sample A in the temperature range from 10 K to 35 K (Figure 14a). Previous research [39] has suggested that *B*_irr_ values depend on the pinning centers. Our study supports this, as we observed an increase in pinning center density after the HIP process, leading to improved *B*_irr_ performance due to the increased density of pinning centers. It was observed that *B*_c2_ is similar for the samples heated at low and high isostatic pressures (Figure 14b). *B*_c2_ is dependent on the mean free path and superconducting coherence length [40]; therefore, the HIP process does not affect these parameters.

## 4. Conclusions

Our study showed that the Mg coating method can significantly reduce the reactions of B with the Fe sheath. The shape, uniformity, and continuity of the layered structure (cracks, gaps) depend on the homogeneity of the B layer before the synthesis reaction. Additionally, studies show that the formation of a layered structure depends on the annealing temperature (in the liquid or solid-state), isostatic pressure, type of boron, and density of layer B before the synthesis reaction. On the basis of tests performed by using an optical microscope and software (Kameram CMOS), we observe that the inner parts of the layered structure possess minimal porosity, while greater porosity is present externally. Moreover, these tests indicate that high porosity occurred at the interlayer connection region and was accompanied by a small number of intergrain connections. Moreover, studies have shown that the layered structure of a sample heat treated under low isostatic pressure has large voids, a heterogeneous layered structure, and high porosity. However, the layered structure of the sample heat treated under high isostatic pressure has superconducting MgB_2_ material, pure B, pure Mg, and high-density layers with excess Mg. Additionally, the HIP process leads to the formation of high-field pinning centers in PIT MgB_2_ wires made using the Mg coating technique.

## Figures and Tables

**Table 1:** Heat treatment processing parameters of undoped MgB_2_ wires.

Sample No.	Pressure [MPa]	Annealing Temperature [°C]	Annealing Time [min]
A	0.1	700	40
B	1100	700	40

## Data Availability

Data are contained within the article.
